# Genetics of autism spectrum disorder & BDNF gene

**DOI:** 10.1186/1755-8166-7-S1-O3

**Published:** 2014-01-21

**Authors:** Usha P Dave

**Affiliations:** 1 Medical Geneticist & Neuroscientist, Mumbai, India

## 

Autism is a group of complex neurodevelopmental disorders which manifests problems with social interaction, language, communication and behavior deficits like stereotype and repetitive activities. Autism prevalence rate in last one decade has shown astonishing level of increase from 1 per 10,000 to 1 in 110 children in USA (CDC, 2007). Various environmental & genetic factors or combination are suggested as contributing factors in this clinically diagnosed neurobehaviour syndrome. However, similar data on prevalence are scarce in India. The etiology of autism still remains unknown, with many factors implicated in the development of autism phenotype. Autism Spectrum Disorder (ASD) is evaluated by a clinical psychologist using DSMR- IV criteria & may manifest mild to severe autistic features, clinical symptoms & low to high intellectual functioning. There are few well characterized genetic/ metabolic conditions (e.g. Rett Syndrome, Tuberous sclerosis, PKU) and chromosomal syndromes (e.g. Fragile-X, Angelman and Prader willi ) where autism has frequently associated features. Multiple genes are thought to be involved in the pathogenesis, but no evidence involving any one particular gene. The recent research focus is also on epigenetic mechanisms operating in complex autism.

Brain Derived Neurotrophic Factor (BDNF) is a neurotophin in the mammalian Central Nervous System & important in neuronal survival, neurogenesis, and synaptic plasticity. BDNF gene is located on chromosome 11. In humans, Val66Met is probably the most investigated SNP of the *BDNF* gene. Since BDNF readily crosses the Blood-Brain-Barrier, the serum concentrations correlate directly to brain concentration, therefore plasma studies of BDNF are thought to accurately reflect CNS concentration. The significance of serum BDNF in ASD to explore its precise role in pathogenesis of ASD and therapeutic relevance is increased with the evidence of BDNF linked with autism. A genetically heterogeneous population of India where consanguinity and endogamous marriages are prevalent genetic risk factors, ASD is a challenge. An attempt is made to study BDNF level & mutations in correlation with the severity of neurobehavioral deficits in Indian patients with ASD & mental retardation. This will be discussed in the light of current scenario.

